# Ovotransferrin in Foods: Digestive Stability, Cross-Matrix Interactions, and Targeted Applications

**DOI:** 10.3390/foods15101673

**Published:** 2026-05-11

**Authors:** Jingyi Zhang, Shujie Chen, Anjia Huang, Xue Zhao, Juan Chen, Yinlong Lian, Chenggang Cai

**Affiliations:** School of Biological and Chemical Engineering, Zhejiang University of Science and Technology, Hangzhou 310023, China; 222403857027@zust.edu.cn (J.Z.); 212403817018@zust.edu.cn (S.C.); 212403817032@zust.edu.cn (A.H.); 222503855005@zust.edu.cn (X.Z.); 222503860009@zust.edu.cn (J.C.); 222503860032@zust.edu.cn (Y.L.)

**Keywords:** ovotransferrin, egg white protein, digestive stability, food matrix interaction, delivery system, natural preservation, functional foods

## Abstract

Ovotransferrin (OVT), a major iron binding glycoprotein in egg white, is increasingly studied as a multifunctional ingredient for food preservation, mineral delivery, and colloidal design. This review critically evaluates how native structure, iron saturation, thermal history, glycation, phosphorylation, fibrillation, and interactions with proteins, polysaccharides, polyphenols, and lipid interfaces influence or determine OVT behavior during processing and gastrointestinal digestion. Rather than defining digestive stability simply as resistance to proteolysis, the review compares how processing routes reshape protease accessibility, peptide release, residual allergenic risk, and the persistence of antimicrobial or antioxidant functions. Particular emphasis is placed on cross-matrix interactions because OVT rarely acts as an isolated purified protein in practical formulations; its performance depends on pH, ionic strength, competing ligands, and the architecture of emulsified, coated, or liquid food systems. The available literature indicates that the most mature application space is multifunctional food system design, including preservation-oriented coatings, Pickering-type emulsions, oleogel-associated systems, and liquid food delivery platforms. Broader industrial applications will require standardized reporting of apo/holo state, processing history, digestion models, real food validation, sensory consequences, and allergenicity. To reduce overinterpretation, the present synthesis prioritizes primary studies and weighs model food or real food validation more heavily than mechanistic or preclinical evidence when discussing application readiness. Overall, OVT should be regarded as a promising but context-dependent protein platform whose value lies in coupling bioactivity with techno-functionality rather than in any single universal health claim. Methodological transparency is further supported by explicit database sources, reproducible search blocks, inclusion/exclusion rules, and a structured quality-appraisal and evidence tiering framework.

## 1. Introduction

OVT, also known as conalbumin, accounts for approximately 12–13% of total hen egg white protein [1,2,3] and has long attracted attention because its metal binding capacity gives rise to antimicrobial, antioxidant, and mineral handling functions that are unusually relevant to food systems. Recent research has widened this interest from classical antimicrobial action [4] to more formulation-oriented questions, including whether OVT can serve as a carrier [2], interfacial stabilizer [5], fibril forming protein [6], or multifunctional ingredient in clean-label foods [3]. This shift matters because OVT is no longer discussed only as a purified egg white fraction; it is increasingly evaluated as a design element in preservation, emulsion structuring, and nutrient delivery.

**Figure 1:**
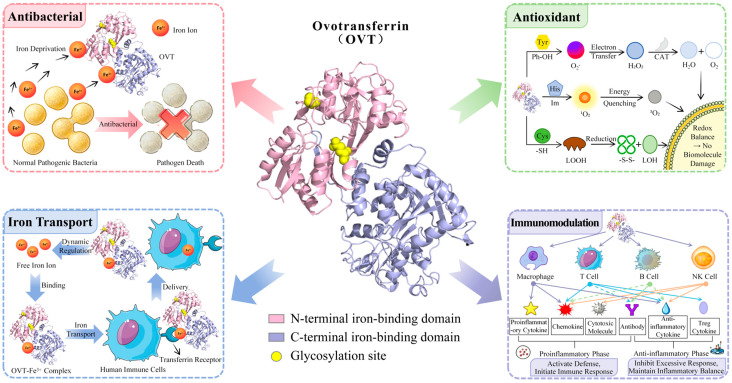
Schematic illustration of the molecular structure and representative core functional mechanisms of ovotransferrin. The scheme summarizes the bilobal Fe^3+^-binding structure of OVT and links structural determinants (apo/holo state, glycosylation, disulfide-rich conformation, and protease-responsive regions) with antibacterial, antioxidant, iron transport, and immunomodulatory functions. Abbreviations and symbols: CAT, catalase; C-terminal, carboxyl terminal; Cys, cysteine; Fe^3+^, ferric iron; H_2_O, water; H_2_O_2_, hydrogen peroxide; His, histidine; Im, imidazole; LOH, lipid alcohol; LOOH, lipid hydroperoxide; N-terminal, amino terminal; NK cell, natural killer cell; O_2_, molecular oxygen; O_2_^−^, superoxide anion; OVT, ovotransferrin; Ph-OH, phenolic hydroxyl group; Treg, regulatory T cell; Tyr, tyrosine; ^1^O_2_, singlet oxygen; ^3^O_2_, triplet oxygen; -SH, sulfhydryl group; -S-S-, disulfide bond.

Structurally, OVT is a disulfide-rich glycoprotein of about 76–78 kDa [1,2,3] with two homologous lobes that coordinate ferric iron in the presence of synergistic anions, such as carbonate [7,8]. This bilobal architecture helps explain why iron saturation, pH, ionic strength, and thermal history strongly influence conformational stability and activity. At the same time, these same variables complicate comparison across studies. Apo- and holo-OVT do not respond identically to heating or fibrillation [9,10]; glycation or phosphorylation may preserve some functions while altering digestion [11,12]; and association with polysaccharides, polyphenols, or other proteins can either protect or mask functionality depending on matrix composition [13,14,15,16]. These structure–function relationships are summarized schematically in Figure 1, which links the bilobal Fe^3+^-binding architecture of OVT with the major structural determinants and representative food-relevant bioactivities discussed in this review.

The aim of this review is, therefore, not to catalogue every reported OVT activity but to compare where the evidence is robust, where it is conditional on model design, and where industrial application in real foods remains premature. Unlike recent broad OVT overviews that summarize structure, general bioactivity, and industrial potential [2,3,6,17], the present review focuses specifically on digestive stability, cross-matrix interactions, and application readiness in food systems. Three questions guide the discussion: (i) which structural variables must be reported before digestion or functionality data can be meaningfully compared; (ii) how cross-matrix interactions redirect OVT behavior during processing and gastrointestinal transit; and (iii) which application claims are supported by model systems, real food validation, or only preclinical evidence. This foods-oriented, evidence-graded approach is necessary because the OVT literature combines purified protein studies [2,6], simulated digestion models [18], cell experiments and animal studies [3,4], and formulation papers [18,19,20] that are not equally informative for food sector deployment.

Table 1 summarizes the structural features that most consistently explain food-relevant OVT behavior. Its purpose is not merely descriptive. Instead, it identifies the variables most likely to account for differences between studies and highlights the structural states that are directly relevant to formulation design, digestion, and safety assessment.

As Table 1 indicates, the most informative descriptors are not simply molecular weight or abundance but apo/holo state, thermal history, surface charge environment, and the presence of protease-responsive or interaction-prone regions. These descriptors recur throughout the literature on digestion, cross-matrix interactions, and applications and should be reported as essential experimental context rather than as optional detail.

To improve methodological transparency, this critical review primarily considered studies published from 2014 onward while retaining a limited number of earlier landmark papers needed to define OVT structure, metal binding behavior, and classical antimicrobial mechanisms. Priority was given to studies on purified OVT [2,3,6], simulated gastrointestinal digestion [8,9,10,15,16,18], cell or animal validation [22,23,24,25], model food systems [26], and, where available, real food verification. The review scope is intentionally centered on digestive stability, cross-matrix interactions, and food application readiness rather than on broad egg protein nutrition claims. Additional comparative background on targeted delivery [27], comparative protein carrier design [28,29], protein digestion comparators [30,31], egg white gels [32], iron homeostasis framing [33,34], and interfacial film behavior [35] was retained only to contextualize formulation logic and was not used as primary support for OVT-specific judgments. Likewise, the broader literature on ovotransferrin-derived peptides [36,37,38,39,40,41,42], heat/allergen processing [43,44,45,46], fibril modification [47,48,49,50], and egg protein valorization [51,52,53,54] was kept as peripheral context rather than claim-defining evidence for food application readiness [42].

### 1.1. Review Scope, Literature Search Strategy, and Evidence Framework

This narrative critical review surveyed the Web of Science Core Collection, Scopus, PubMed, and Google Scholar using combinations of the terms “ovotransferrin”, “conalbumin”, “digestion”, “gastrointestinal”, “iron saturation”, “glycation”, “phosphorylation”, “fibril*”, “polyphenol*”, “polysaccharide*”, “emulsion*”, “Pickering”, “delivery”, “coating”, “allergen*”, “cross-matrix interaction*”, and “food matrix”. Emphasis was placed on studies published from 2014 onward, while earlier landmark papers were retained when needed to define OVT structure, metal binding, or classical antimicrobial mechanisms. Eligible papers had to contain direct information relevant to food processing, digestion, cross-matrix interactions, or food-oriented applications; conference abstracts, duplicate records, and papers with insufficient experimental description were not used as claim-defining evidence. The final literature search was completed on 01 April 2026, with no language restrictions applied. This cutoff date was selected to cover the 2026 ahead-of-print and early-volume studies already cited in the reference list of this review.

To make the search process reproducible, the core Boolean structure was (“ovotransferrin” OR “conalbumin”) AND one or more topic blocks, including (“digestion” OR “gastrointestinal” OR “INFOGEST” OR “proteolysis”), (“iron saturation” OR “apo” OR “holo” OR “metal binding”), (“glycation” OR “phosphorylation” OR “fibril*” OR “aggregation”), (“polyphenol*” OR “polysaccharide*” OR “emulsion*” OR “Pickering” OR “coating”), and (“allergen*” OR “safety” OR “regulation” OR “scale-up”). Titles and abstracts were screened first, and full texts were then assessed when the article could support an OVT-specific statement on processing, digestion, cross-matrix interactions, or food applications.

Studies were then appraised qualitatively according to four questions: whether the OVT state and pretreatment were clearly described, whether the digestion or matrix model was appropriate for the stated claim, whether the endpoint measured stability or functionality directly, and how closely the work approached practical food use. On this basis, evidence was assigned to the structured hierarchy specified below before application readiness conclusions were drawn.

The inclusion criteria were (i) direct use or characterization of OVT or clearly identified OVT-derived peptides; (ii) reporting of processing, digestion, interaction, bioactivity, safety, or food application endpoints relevant to the review questions; and (iii) sufficient methodological detail to interpret the tested OVT state, matrix, or model system. Exclusion criteria were (i) records focused only on whole egg nutrition without OVT-specific data; (ii) conference abstracts, patents, non-peer-reviewed sources, or duplicated reports; and (iii) studies in which the experimental design did not allow the claimed outcome to be attributed to OVT. Study quality was classified as high, moderate, or low confidence according to the clarity of OVT characterization, model appropriateness, endpoint relevance, and proximity to practical food use; low-confidence studies were retained only as background context and not as primary evidence for application readiness conclusions.

The evidence hierarchy was applied before drawing application conclusions: Level 1 = purified-protein mechanistic evidence; Level 2 = simulated digestion, cell, or simplified biological evidence; Level 3 = model food or formulation evidence; and Level 4 = real food shelf-life, animal in vivo, or human evidence directly supporting the proposed use. For food design conclusions, Level 3 or Level 4 food matrix evidence was considered more decisive than Level 1 or Level 2 mechanistic plausibility. When animal or cell studies reached a higher biological tier but lacked formulation validation, the claim was labeled as preclinical rather than application-ready. The recommended descriptors for reporting OVT digestion studies and interpreting comparability were summaried in Table 2.

## 2. Digestion, Metabolism, and Activity Preservation Mechanisms

### 2.1. Gastrointestinal Fate of OVT: What Is Established and What Remains Uncertain

The gastrointestinal fate of OVT is often described in overly simple terms, as if the protein were either digested or not. The available evidence supports a more nuanced interpretation. Native OVT is destabilized under gastric acidity [3,6] and then further hydrolyzed during the intestinal phase [8,21], but the meaning of this hydrolysis depends on the endpoint being measured: disappearance of intact protein [6], generation of peptides [9,10,15,16], retention of iron binding fragments [8], persistence of allergenic epitopes [18], or preservation of pre-absorption antimicrobial activity [3,21]. For food design, digestive stability is, therefore, better understood as a controllable digestion pattern than as absolute resistance to breakdown.

The comparability of digestion studies remains limited because the literature uses different enzyme systems, residence times, pH conditions, matrix backgrounds, and analytical endpoints. Some studies prioritize sodium dodecyl sulfate–polyacrylamide gel electrophoresis (SDS-PAGE) disappearance [6,9], whereas others evaluate peptide fingerprints [15,16], antioxidant capacity after digestion [3], epithelial transport [23], or allergenicity markers [18]. These endpoints are not interchangeable. Apparent disagreements in whether OVT fibrils are “more stable” [9,10,15,16] or iron-bound OVT is “less digestible” [8] often reflect different operational definitions of “stability” rather than a true contradiction. Such claims should, therefore, be interpreted as protocol-dependent shifts in protease accessibility, peptide release, or epitope persistence—not as universal rankings that apply across all foods [3,4,6].

The major inconsistencies in digestion behavior can be organized into three recurring patterns. First, fibrillar or aggregated OVT may appear more resistant by SDS-PAGE while still releasing distinct peptide populations that change antioxidant, antimicrobial, or immunological readouts. Second, iron saturation can reduce protease accessibility by stabilizing selected conformations, but this effect may be weakened under acidic gastric conditions or in matrices containing citrate, phosphate, and competing proteins. Third, covalent or non-covalent complexes with phenolics and polysaccharides can either shield cleavage sites or create new aggregation interfaces, so their influence depends on the assembly route rather than on the presence of the partner molecule alone.

Iron saturation is one of the most important variables in this context. Recent structure–activity work shows that different iron saturation levels alter OVT conformation, thermal behavior, fibrillation tendency, and peptide release during digestion [8]. Nevertheless, apo/holo state should not be treated as a catch-all explanation for every discrepancy in the literature. Digestive outcome is also shaped by prior aggregation, fibrillation route, covalent modification, association with polyphenols or polyelectrolytes, and whether digestion occurs in dilute buffer or in a more competitive matrix. Studies that do not report iron saturation, pretreatment history, and digestion background, therefore, remain difficult to compare directly.

A second issue that deserves equal weight is the distinction between nutritional and safety endpoints. Slower digestion is not automatically desirable if it preserves allergenic epitopes, and faster hydrolysis is not inherently detrimental if it generates peptides with useful antioxidant or antimicrobial activity at the intended stage of digestion. Recent integrated in vitro/in vivo evidence [18] showing residual allergenic risk after digestion strengthens the argument that modified digestion kinetics should be evaluated not only for retained functionality but also for their safety implications. Earlier heat treatment studies [44] and allergen-processing studies [41] likewise indicate that structural perturbation can shift immunoglobulin E (IgE) reactivity without uniformly abolishing risk, reinforcing the need to interpret slower digestion or residual intactness as a safety question as well as a functionality question.

### 2.2. Processing-Induced Changes Before Digestion

Processing history predetermines digestive behavior because it reshapes the balance between unfolding, aggregation, interfacial adsorption, and protease accessibility before OVT even enters the gastrointestinal environment. Heating is especially important because OVT is among the more heat-sensitive egg white proteins, and its response depends strongly on pH [7], ionic conditions [4,11], and the presence of stabilizing or destabilizing co-solutes [8,55,56]. Mild pasteurization-like treatment may only partially perturb the native fold, whereas treatment near the isoelectric region or under poorly buffered conditions can promote aggregation, loss of iron binding function, and altered peptide release after digestion.

Not all processing routes generate comparable intermediates. Phosphorylation mainly shifts charge distribution and thermal tolerance [11], controlled glycation changes surface hydrophobicity and can redirect fibrillation [12,18], and enzymatic or acid-driven fibrillation produces elongated supramolecular assemblies whose digestive accessibility differs from that of amorphous heat aggregates [9,15,16,57]. Freeze–thaw history, ultrasound-assisted assembly, and interaction with lysozyme or polyanions further complicate interpretation. For that reason, broad claims such as “processing improves OVT stability” are not very informative unless they specify which property is stabilized [7,11], under what matrix conditions [55,56,58], and at what trade-off to digestibility, clarity, flavor neutrality, or processability [59].

Table 3, therefore, categorizes the reported strategies not simply by the method used but by the type of protection they offer and the specific food problem they are most likely to address. This distinction is important, as a processing route that better preserves native conformation under heating may differ from one that enhances interfacial stabilization or modulates peptide release during digestion.

### 2.3. Activity Preservation Strategies from Processing to Digestion

Current activity preservation strategies can be grouped into three broad categories: physical shielding, molecular modification, and supramolecular assembly. Physical shielding includes particles, capsules, and interfacial layers [61,62,67,69] that limit direct environmental exposure. Molecular modification changes the protein itself through phosphorylation, glycation, or related reactions [11,12,18]. Supramolecular assembly includes fibrils [9,16,25,58], heteroprotein complexes [59,64,65], coacervates [20], and Pickering-type particles formed with other biopolymers [19,61,70,71]. This classification is useful because each route preserves function through a different mechanism and should, therefore, be matched to a different formulation objective.

Application context is decisive here. A beverage or liquid food system typically prioritizes colloidal stability, low sedimentation, acceptable viscosity, and tolerance to acid, salts, or storage-induced changes. A coating or emulsion system instead prioritizes interfacial adsorption, droplet stabilization, controlled release, and retention of hydrophobic cargo. Fibrillar OVT and OVT–gum arabic assemblies appear especially promising for Pickering-type interfaces [62,66,67] and oleogel-associated systems [22,25,61,69], whereas phosphorylation is more directly relevant when the formulation goal is to retain a more native-like protein under moderate heat [7,11,26]. Controlled glycation [12,18] and polyphenol association [12,15,16,71] can improve antioxidant or interfacial properties, but they also reshape digestibility and should not be presented as uniformly beneficial without matrix-specific validation.

**Figure 2:**
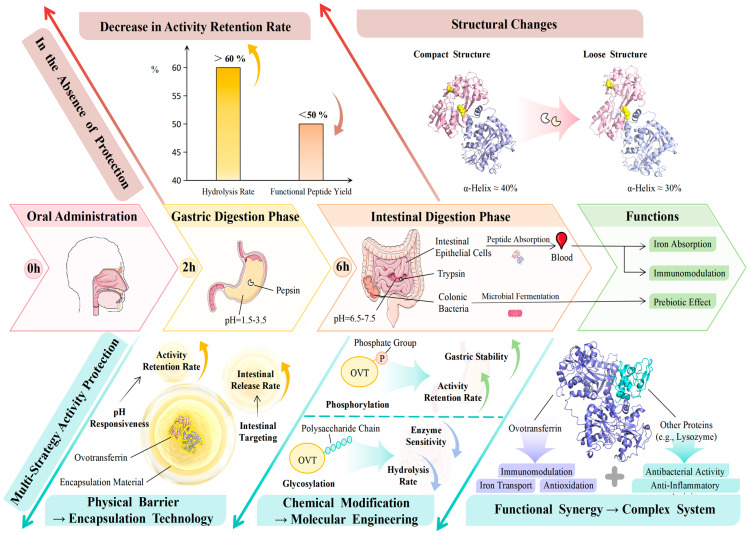
Conceptual scheme of OVT digestion, metabolic fate, and major activity preservation routes during processing and gastrointestinal transit. The diagram organizes the sequence from processing exposure to gastric and intestinal digestion, indicating where physical shielding, molecular modification, and supramolecular assembly may preserve, redirect, or reduce OVT functionality. Abbreviations: OVT, ovotransferrin.

Figure 2 presents these preservation routes as a conceptual sequence that links processing exposure, structure formation, and gastrointestinal fate. It should be viewed as an evidence-guided framework for identifying where functionality is lost, preserved, or redirected—not as a universal mechanism that applies equally to all formulations.

Table 3 compares representative strategies by asking not only what each route does to OVT but also which type of food problem it addresses and where current support remains weak. In this way, the table distinguishes structurally persuasive strategies from those that are closer to formulation readiness. Taken together, Table 3 suggests that the strongest current evidence supports moderate thermal stabilization by phosphorylation [7,11] and improved interfacial or cargo protection performance by fibrils and polysaccharide-associated assemblies [22,61,62,69]. By contrast, claims of precise intestinal targeting or broadly preserved bioactivity after oral delivery remain much less mature and are still mainly supported by model system evidence.

### 2.4. How Comparable Are Current Digestion Studies?

A recurring limitation in the OVT literature is that digestion, processing, and functionality are often studied in separate experimental silos. One paper may compare apo- and holo-OVT under standardized digestion, another may examine fibrils in an emulsion model, and a third may report antibacterial activity after hydrolysis. Each design is valid for its own question, but direct comparison is restricted because the controlled variable, matrix background, and endpoint are different. This helps explain why the same processing route may appear to improve OVT “stability” in one study yet reduce it in another.

To improve comparability, at least five descriptors should be reported consistently: OVT source and purity, iron saturation state, pretreatment history, matrix composition during digestion, and the endpoint used to define retained function or stability. Without these descriptors, apo/holo effects [8] can easily be overinterpreted, and route-specific behaviors of fibrils [9,10], heat aggregates [18], or polyphenol complexes [15,16] may be mistaken for general properties of OVT itself.

From a food perspective, comparability also requires moving beyond enzyme-only model systems. Simulated digestion remains valuable for mechanism building, but real-food relevance depends on whether the same trends survive in the presence of competing proteins, emulsified lipids, salts, sugars, and storage-induced structural change. The same trend observed in dilute buffer may not persist in emulsified foods [66,67,69], protein-rich foods, or acidified foods [22,24,25,61], where competitive adsorption, ionic screening, and phase partitioning can alter both substrate accessibility and peptide release [4]. Digestion studies embedded in actual formulation backgrounds are, therefore, likely to be more informative for industrial application than additional work conducted only in dilute buffer.

## 3. Interaction Mechanisms with Food Matrix Components

OVT rarely functions as an isolated molecule once it is placed in a food matrix. Its performance is redirected by competitive ligands, pH-dependent charge balance, interfacial adsorption, and association with other biopolymers. The most useful way to review OVT cross-matrix interactions is, therefore, not to list every reported partner but to compare which interaction classes are mechanistically robust [4,8,72], which are highly condition-dependent [3,15,23] and which have already been exploited in food-relevant microstructures [20,61,66,67].

### 3.1. Metal Ions

Metal binding is the defining interaction class for OVT. Ferric iron coordination underlies the classical iron withholding mechanism associated with antimicrobial action [1,4,72] and also explains why OVT is attractive as a putative food-compatible iron carrier [3,23]. Recent work further shows that iron saturation changes not only the biological interpretation but also physicochemical behavior, including thermal response, fibrillation tendency, and digestion pattern [8]. In practical fortified systems, however, Fe^3+^-OVT behavior must be interpreted together with co-solute composition because citrate, phosphate, salts, and pH shifts can change iron speciation, competitive binding, and, therefore, the apparent benefit of OVT-based sequestration or delivery. This is one reason why data from dilute buffers should not be extrapolated directly to beverages, emulsions, or protein-rich foods.

Evidence for zinc ion (Zn^2+^) and other non-iron metals is mechanistically informative but much less ready for direct food application. Available data indicate that alternative metal ions can alter antibacterial behavior [73], yet the magnitude and even the direction of that effect depend on pH, competing ligands, and ionic environment [8,72]. In complex foods, phosphates, citrate, salts, and polyphenols may all compete for binding or modify speciation. Metal-ion effects beyond Fe^3+^ should, therefore, be treated as condition-specific modulators rather than as general formulation tools.

### 3.2. Polyphenols and Other Bioactive Small Molecules

Polyphenols are among the most actively studied classes of OVT partners because they can simultaneously change conformation, antioxidant behavior, fibrillation kinetics, and digestion. Catechin [14], gallic acid [15], and chlorogenic acid-related systems [74] show that both covalent and non-covalent association can modify surface hydrophobicity, redox behavior, and protease accessibility [16,71]. These results are promising, especially for co-delivery concepts in which the protein protects an oxidation-sensitive phytochemical or provides a structured carrier interface. A key formulation question, however, is whether the phenolic partner stabilizes OVT without causing unacceptable browning, haze, or astringency; these trade-offs are still reported less consistently than particle size or antioxidant readouts.

Yet the benefits of polyphenol binding are clearly context-dependent. A stronger association is not always advantageous if it blocks sites important for metal binding, drives excessive insolubilization, or compromises optical or sensory properties. In addition, many reported advantages are demonstrated in model dispersions or under controlled assembly conditions rather than in finished foods. The most informative studies are, therefore, those that track both colloidal or interfacial performance and digestion outcomes under the same assembly conditions because they make it easier to distinguish functional protection from simple insolubilization. Polyphenol-associated OVT systems are, therefore, best described as tunable platforms whose value depends on matching small-molecule chemistry to the intended matrix, storage condition, and digestive endpoint.

### 3.3. Polysaccharides, Interfaces, and Complex Food Microstructures

Interactions with polysaccharides and interfaces currently provide some of the most direct routes to food applications. OVT fibrils [22,61,62,73], OVT–gum arabic complexes [66,67,69], sugar beet pectin complexes [13], and chitosan-associated systems [20,70] have been used to stabilize emulsions, build oleogel-derived structures, and protect hydrophobic cargos, such as hesperidin or curcumin. In these systems, OVT acts not only as a bioactive protein but also as a structural component that helps define particle architecture, interfacial packing, or rheology. The strongest studies in this category are those that connect particle architecture to interfacial behavior, storage stability, and post-digestion cargo retention within one experimental design because this makes the food relevance of the assembly route much clearer.

The practical advantage of this interaction class is that performance can be measured using metrics that are directly relevant to practical application, including droplet stability, retention of encapsulated actives, rheology, and storage robustness. The limitations are equally clear. Most studies still rely on simplified emulsions, oleogel-derived model systems, or beverage-like dispersions. Real foods introduce salts, sugars, native proteins, flavor compounds, and process histories [4] that may weaken electrostatic assembly or alter interfacial behavior. Interface performance in model emulsions [22,66,67,69] does not automatically translate to full formulations containing salts, sugars, flavor compounds, and processing-induced competitive adsorption [20,61]. For that reason, matrix-enabled performance should be demonstrated in systems that retain at least the major compositional constraints of the target food, not inferred only from simplified aqueous models. Promising polysaccharide or interface strategies should, therefore, be described as matrix-enabled rather than matrix-independent.

Table 4 maps the main OVT cross-matrix interaction classes to likely consequences and candidate food uses. Its function is not simply to catalogue partners but to distinguish robust interaction logic from early-stage associative findings and to indicate where experimental support is already architecture-specific.

### 3.4. Which Interaction Strategy Fits Which Food Matrix?

A more useful formulation question is not whether polyphenol, polysaccharide, fibril, or heteroprotein strategies are optimal in general but which strategy best matches a given matrix. Polyphenol association is attractive when antioxidant co-functionality or phytochemical retention is desired, but it can complicate clarity, color, or astringency in beverages. Polysaccharide association is particularly suitable for aqueous dispersions, emulsions, and coatings in which electrostatic or hydrogen-bonded complexation can be maintained. Fibrillar OVT is especially advantageous where Pickering-like interfacial stabilization or viscosity building is beneficial [22,61,62,67], although it may be less appropriate for products that require a fully native sensory profile [71].

Heteroprotein assemblies, especially with lysozyme, may provide multifunctionality by combining interfacial, foaming, and antimicrobial mechanisms [47,59,63,65]. Their potential appears strongest in foams, aerated systems, or interface-rich formulations, but the workable pH window and process tolerance of these assemblies are not yet broad enough to justify claims of universal applicability. Overall, Table 4 should be read as a matrix-selection guide; it clarifies where interaction chemistry is most promising while also showing that most routes remain supported mainly by model food evidence rather than by commercial-type products.

In this sense, Table 4 contributes more than a list of interaction partners. It clarifies which classes already align with recognizable food architectures and which remain primarily mechanistic. That distinction is essential when assessing whether an OVT-based strategy is approaching practical usefulness or remains at the proof-of-concept stage.

## 4. Targeted Applications for Ovotransferrin in the Food Sector

The application literature on OVT is best interpreted through an evidence-graded lens. Some proposed uses are mainly supported by mechanistic or preclinical data, whereas others are anchored in measurable performance within formulated food systems. The most defensible near-term applications are, therefore, not necessarily the most biologically ambitious ones. For foods, the central question is whether a claimed application is supported at the appropriate level—concept validation [6,75,76], model food testing [21,57,77], or industrially relevant food performance [4,5,23,72].

### 4.1. Natural Preservation and Shelf-Life Extension

Natural preservation remains one of the most attractive OVT application areas because it connects a well-established mechanistic rationale—iron sequestration [4] and, under some conditions, membrane-related effects [72,78]—to the food sector’s need for clean-label microbial control [57,63,77]. Current data support the view that OVT and certain OVT-derived hydrolysates [57,77] can suppress biofilm formation or inhibit selected pathogens in vitro [73]. However, not all hydrolysis routes yield robust preservation activity; single-enzyme hydrolysis [79] has also produced limited or inconsistent antimicrobial outputs, underscoring the need to distinguish peptide-specific evidence from generic hydrolysate claims [45]. The application case becomes stronger when OVT is incorporated into coatings [20,70], emulsion interfaces [22,62,67,69], or composite systems that improve contact efficiency or retention at the food surface.

Even so, preservation claims should remain moderate. In most studies, evidence still comes from broth assays [57], plate tests [77], or simplified model foods [20,62,67,70] rather than from shelf-life studies performed in commercially realistic matrices. Conflicting antimicrobial findings also need to be interpreted carefully. Activity depends on whether the tested material is intact OVT, a hydrolysate, or a thermally assembled form; on the target microorganism and assay medium; and on whether the matrix permits iron withholding or surface contact to remain effective. Broth inhibition, biofilm suppression, and shelf-life protection should, therefore, not be read as interchangeable levels of evidence. The next step is not another isolated demonstration of antimicrobial activity but validation under storage, packaging, and sensory conditions relevant to actual foods.

The inconsistent antimicrobial results are, therefore, not surprising. Iron depletion mechanisms are most visible in low-iron broth systems, whereas surface contact or membrane-related effects require sufficient proximity between OVT-based structures and target cells. Hydrolysates add another layer of variability because peptide length, charge, hydrophobicity, and residual iron binding capacity differ among enzymatic routes. Consequently, antimicrobial evidence should be graded separately for broth inhibition, biofilm disruption, coating performance, and verified shelf-life extension in real foods.

### 4.2. Iron Delivery and Mineral-Focused Functional Foods

Iron delivery is arguably the most mechanistically coherent nutrition-oriented application because it builds directly on OVT’s native metal binding function [3,8]. In vitro gastrointestinal [23] and epithelial models [3] suggest that OVT can influence iron availability at absorptive interfaces and may support iron handling in a food-compatible form. This makes OVT scientifically interesting for mineral-focused functional foods, especially when iron compatibility must be coupled with broader protein functionality.

However, this application should still be framed cautiously. The strongest support remains at the level of in vitro digestion [8,23] and transport-related models [3]; support is far weaker for direct efficacy claims in specific populations or finished fortified foods. In particular, extension to infant, clinical, or other special population products would require much stronger evidence on allergenicity [18,43], dose, regulatory acceptability [80], and performance in real formulations.

### 4.3. Gastrointestinal and Immunological Functionality

Reports of anti-inflammatory, barrier-protective, or immune-modulating effects in cultured cells [75,81] and murine models [5,6,76,82] are scientifically valuable because they suggest that OVT or OVT-derived fragments may act beyond simple nutrient delivery. These studies also show why digestion should not be viewed only as a loss pathway; under some conditions, hydrolysis may generate peptides with retained or redirected activity. At the same time, the peptide and hydrolysate literature remains heterogeneous, with some studies reporting defined anti-inflammatory, peptide-mediated, or osteogenic activities [38,83,84] while others show limited functional gains after single-enzyme hydrolysis [45,54], so food positioning claims should remain explicitly evidence-tiered.

Nevertheless, this application domain remains preclinical. It is reasonable to discuss gut-focused or inflammation-related functionality as a promising research direction, but not as a validated food claim. The current literature does not justify strong positioning of OVT for postoperative nutrition, geriatric nutrition, or medical foods without a much clearer bridge between mechanism, dose, safety, sensory feasibility, and product format.

### 4.4. Structuring and Delivery Roles in Emulsified or Liquid Foods

Structuring and delivery roles in emulsified or liquid foods appear to be the most immediate route toward industrial relevance. Fibrillar OVT [22,24,25,61], OVT–gum arabic complexes [66,67,69], OVT particles [13,62], and OVT–lysozyme assemblies [20,59,64,77] have already been used to improve interfacial stability, cargo retention, and rheological behavior in Pickering-type emulsions, oleogel-associated systems, and fortified liquid foods. Unlike many bioactivity-focused claims, these outcomes can be quantified through directly relevant food engineering endpoints, which makes the evidence base more actionable for formulation development.

Application readiness was interpreted according to the four-level evidence hierarchy defined in Section 1.1; because formulation claims and health positioning claims are not validated in the same way, real food studies were weighted most heavily for food design conclusions, whereas biological models were treated as supportive but not equivalent evidence.

Table 5 makes this distinction explicit. Its value lies not simply in listing possible uses but in separating applications already supported by food physics data from those that remain driven mainly by biological plausibility.

Accordingly, Table 5 functions as a readiness map linking each application to its dominant evidence tier and its principal barrier to practical deployment.

Viewed in this way, the near-term value of OVT lies less in positioning it as a clinically established bioactive and more in using it as a multifunctional structuring ingredient in emulsified, coated, and liquid food systems. That positioning better matches both the current evidence base and the readership of Foods.

## 5. Industrialization Challenges and Future Research Directions

The industrial promise of OVT lies in its unusual combination of bioactivity and techno-functionality, but industrial deployment remains constrained by several unresolved bottlenecks. The first is ingredient standardization. Recent work has improved extraction and fractionation routes [56,85], yet large-scale deployment still requires tighter control of purity, iron saturation state, batch-to-batch variability, and processing history before OVT can be treated as a reliable formulation ingredient rather than a variable egg-derived fraction [80]. Compared with more established carrier proteins, such as whey proteins or broader food-protein ingredients, OVT currently has a less mature supply chain and a smaller body of scale-up, cost, and manufacturing compatibility data [21,28,56,68]. Its practical entry point may, therefore, be premium or function-specific systems in which iron binding, antimicrobial contribution, and interfacial performance are all needed simultaneously.

From a scalability perspective, OVT differs from high-volume carrier proteins such as whey protein, casein, soy protein, pea protein, or gelatin. These alternatives have broader supply chains, lower cost familiarity, and more established regulatory and processing histories, whereas OVT offers a narrower but more distinctive functional profile based on iron binding, egg-derived antimicrobial activity, and interfacial assembly. This comparison suggests that OVT is unlikely to replace commodity proteins in general structuring applications; its more realistic industrial role is in value-added formulations where several of its functions are needed simultaneously and where egg allergen labeling is already acceptable.

A second bottleneck is the gap between model system success and real food performance. Many reported OVT advantages have been demonstrated in simplified buffers [4], emulsions [11,78], or digestion models [8,9], but far fewer studies test whether the same benefits survive pasteurization, drying, acidification, high-shear processing, storage, or consumer preparation [26,56]. For a protein whose behavior is highly context-dependent, real food validation is not a late-stage refinement; it is central to assessing whether a proposed function is meaningful at all.

Allergenicity and labeling represent a third bottleneck and should be discussed alongside, not after, functionality. OVT is a recognized egg allergen [43,80], and recent evidence indicates that digestion does not automatically eliminate residual allergenic risk [18]. This issue becomes especially important whenever OVT is proposed for vulnerable populations or positioned as a value-added bioactive ingredient. For real food applications, the central risk–benefit question is whether the gain in preservation support, mineral handling, or delivery performance justifies the use of a declared egg allergen in the intended product and consumer group. In practice, egg allergen labeling, regional regulatory expectations for functional claims, and the need to substantiate dose–response benefits may narrow the most realistic product categories for OVT deployment [43,80]. Any industrial narrative that emphasizes preserved functionality without parallel consideration of allergenicity, dose, and regulatory status remains incomplete.

A practical risk–benefit assessment should, therefore, be product-specific. In egg-containing foods, OVT may add preservation support or delivery functionality without introducing a new allergen category, although processing-induced changes in epitope exposure still require verification. In allergen-free, infant, clinical, or school food products, the same functionality may not justify the labeling and exposure burden unless clear dose–response benefits, residual allergen data, and consumer risk controls are available. This distinction helps prevent the functional advantages of OVT from being overstated in product categories where allergen avoidance is a primary requirement.

A fourth challenge is sensory and process compatibility. Fibrils, polyphenol complexes, or heteroprotein assemblies may improve interfacial performance yet still fail in practice if they introduce haze, excessive viscosity, off-color, flavor interactions, or poor stability in salt- and sugar-rich foods. Future studies should, therefore, report not only particle size, zeta potential, and in vitro release but also appearance, mouthfeel, storage behavior, and compatibility with realistic manufacturing conditions.

Regulatory feasibility also depends on the type of claim being made. Use of OVT as a technological ingredient for texture, interface stabilization, or preservation support may require different substantiation than claims of improved iron status, gut protection, or immune modulation. Future application studies should, therefore, define intended use, dose range, target consumers, labeling pathway, and comparator protein at the beginning of the experimental design rather than adding regulatory discussion only after functional effects have been observed.

Looking forward, the most productive research agenda is one that integrates structure-resolved chemistry with application-oriented food testing. Priority areas include head-to-head comparisons of native, iron-saturated, phosphorylated, glycated, and fibrillar OVT [4,8,72,80]; digestion studies conducted in real formulation backgrounds [18]; validation of interfacial systems after storage and processing [3,13]; and coordinated assessment of allergenicity, labeling, and regulatory fit [80]. Progress on these fronts would move the field beyond proof-of-concept multifunctionality toward reproducible ingredient design.

Accordingly, four near-term priorities should be emphasized. First, digestion studies need standardized reporting of apo/holo state [8], pretreatment history, matrix background, and endpoint definition [18]. Second, preservation support [11], emulsion [22,24,25], and liquid food claims [26] should be re-tested in real or semi-real food systems rather than only in dilute models. Third, digestibility, residual allergenicity [43,80], sensory compatibility, and process robustness should be evaluated together instead of as disconnected endpoints. Fourth, special population or gut health positioning should remain a later-stage objective until these deployment gaps are narrowed.

## 6. Conclusions

OVT should be regarded as a promising but highly context-dependent food protein platform. Its value derives not from a single dominant function but from the way iron binding [1,8,23,72], antimicrobial potential [6], oxidative interactions [3,13], digestibility [9,10,15,16], and interfacial behavior can be coupled or redirected through processing and matrix design.

At present, the strongest evidence supports OVT as a multifunctional ingredient for preservation support systems [4,57,72], emulsified delivery structures [3,62,67,69], oleogel-associated or Pickering-type interfaces [22,24,25,61], and selected mineral-focused formulations [23]. By contrast, gut health [75,76,81,82] and special population claims [43] remain biologically interesting but not yet sufficiently validated for strong food application positioning. Continued progress will depend on better comparability across digestion studies, more realistic matrix validation, and earlier integration of allergenicity and regulatory considerations into formulation research. If these requirements are addressed systematically, OVT can advance from a promising egg white bioactive to a more reliable design tool for next-generation food systems.

## Figures and Tables

**Table 1:** Key structural features of OVT and their food-relevant functional implications.

Structural Attribute	What Is Well Supported	Main Food Relevance	References
Relative abundance in egg whites	OVT is one of the major egg white proteins and usually represents about 12–13% of total egg white protein.	Explains why even moderate structural changes in OVT can influence whole egg white functionality and downstream fractionation value.	[1,2,3]
Bilobal iron binding architecture	Each lobe can coordinate ferric iron, and iron saturation measurably alters size, thermal behavior, and digestion pattern.	Supports mineral sequestration, antimicrobial action, and possible iron-delivery applications.	[1,2,3,8]
Disulfide-rich conformation	The native globular fold is stabilized by multiple disulfide bonds and is sensitive to pH- and heat-driven rearrangement.	Relevant to pasteurization tolerance, aggregation, gelation, and retention of ligand binding function.	[1,7,11]
Glycosylation and surface charge	Native glycosylation and charge environment affect intermolecular interactions, fibrillation, and colloidal behavior.	Important for emulsion stabilization, complexation with polysaccharides, and encapsulation design.	[12,13,15,16,17]
Apo/holo state	Iron-free and iron-bound states do not behave identically during heating, fibrillation, or digestion.	Needs to be considered when comparing studies or designing iron-fortified systems.	[7,8,10]
Protease-responsive regions	OVT is susceptible to structural changes and peptide release during gastrointestinal digestion; digestibility depends on prior processing and assembly state.	Directly affects bioactivity retention, peptide generation, and allergenicity assessment.	[6,9,15,16,18,21]

Note: The table summarizes structural descriptors with the strongest relevance to food formulation and digestion comparisons. Abbreviations: OVT, ovotransferrin; apo, iron-free state; holo, iron-bound state.

**Table 2:** Recommended descriptors for reporting OVT digestion studies and interpreting comparability.

Descriptor to Report	Representative Examples	Why It Matters	Risk If Omitted
OVT source and purity	Egg white extract, fractionated preparation, purified OVT	Determines whether coexisting proteins or impurities contribute to the observed behavior	Effects may be misattributed to OVT
Iron saturation state	Apo, partial saturation, holo; ferric loading protocol	Strongly affects conformation, thermal response, fibrillation, and digestion	Apo/holo effects can be conflated with processing
Pretreatment history	Heating, pH shift, freeze–thaw, phosphorylation, glycation, ultrasound	Predetermines aggregation pathway and protease accessibility	Cross-study contradictions become hard to interpret
Assembly state before digestion	Native protein, aggregate, fibril, particle, coacervate, or complex	Structure controls surface exposure, diffusion barriers, and interfacial behavior	“Stability” becomes undefined across formats
Digestion model and matrix background	Static in vitro digestion model developed by the COST Action INFOGEST (INFOGEST-like system), enzyme composition, residence time, buffer vs. emulsion vs. beverage	Determines protease exposure and competitive interactions in realistic foods	Dilute buffer results may be overgeneralized
Readout used to define outcome	SDS-PAGE disappearance, peptide profile, residual epitope, retained antimicrobial/antioxidant function, iron handling	Different endpoints capture different meanings of stability or retained functions	Intactness, peptide release, and function may be conflated

Note: These descriptors are recommended as minimum reporting items for comparing OVT digestion studies rather than as exclusion criteria for the literature survey. Abbreviations: INFOGEST, international consensus static in vitro digestion model developed by the COST Action INFOGEST; OVT, ovotransferrin; SDS-PAGE, sodium dodecyl sulfate–polyacrylamide gel electrophoresis.

**Table 3:** Representative processing and protection strategies affecting OVT stability and digestive behavior.

Strategy	Typical Effect on OVT	Main Advantage	Important Limitation	References
Phosphorylation	Raises pasteurization tolerance and can lessen heat-induced loss of native structure.	Useful when OVT must survive moderate thermal treatment.	Benefit is context-dependent and does not guarantee protection during full digestion.	[7,11]
Controlled glycation	Changes surface hydrophobicity, fibrillation behavior, and emulsifying properties.	Can improve functionality and carrier behavior in food dispersions.	Needs control to avoid excessive browning or poorly characterized Maillard products.	[12,18,60]
Polyanion-assisted stabilization	Dextran sulfate or similar polyanions suppress aggregation under selected conditions.	Promising for buffering heat sensitivity in model systems.	Food-grade applicability, ionic strength dependence, and regulatory fit require evaluation.	[58]
OVT fibrillation	Generates elongated assemblies with altered rheology, digestibility, and carrier performance.	Supports Pickering emulsions, nutrient loading, and liquid food texture design.	Function depends on fibril preparation route and may not mimic native OVT behavior.	[9,22,61,62]
Protein–protein complexation	Complexes with lysozyme modify foaming, interfacial properties, and iron-related antimicrobial cooperation.	Can couple complementary functions in one ingredient system.	Complex coacervation windows are narrow and strongly pH-dependent.	[59,63,64,65]
Polysaccharide association	Pectin, gum arabic, or chitosan can improve colloidal stability and protect cargo.	Adaptable to emulsions, coatings, and oral delivery concepts.	Salt, pH, and matrix competition may weaken electrostatic protection.	[20,61,66,67]
Encapsulation with broader protein carriers	Borrowing general food protein carrier concepts can reduce direct environmental exposure.	Conceptually useful for food-oriented formulation design.	Evidence specific to OVT remains more limited than for whey or plant proteins.	[21,28,32,68]

Note: The table compares representative processing or protection strategies by their main intended protective mechanism and translational limitation. Abbreviations: OVT, ovotransferrin.

**Table 4:** Major classes of OVT cross-matrix interactions relevant to food applications.

Matrix Component	Interaction Mode	Likely Consequence	Application Implication	References
Fe^3+^	Specific metal coordination in native lobes	Alters conformation, thermal behavior, and digestion pattern.	Relevant to iron fortification and antimicrobial iron sequestration.	[3,4,8,72]
Zn^2+^ and other metal ions	Competitive or auxiliary metal binding	May change antibacterial behavior, but outcomes are condition-specific.	Useful mechanistically; requires validation in real foods.	[8,72,73]
Catechin/gallic acid/epigallocatechin gallate (EGCG)	Non-covalent and/or covalent protein–polyphenol association	Changes antioxidant capacity, fibrillation, and digestibility.	Promising for co-delivery of phytochemicals and antioxidant design.	[14,15,71,74]
Chlorogenic acid-related complexes	Small-molecule association assisted by ultrasound or processing	Can modify immunological or physicochemical behavior in model systems.	Potential functional food route, but safety and mechanism need careful evaluation.	[71,74]
Pectin and gum arabic	Electrostatic/hydrogen-bonded complexation with native protein or fibrils	Improves Pickering or oleogel emulsion stability and cargo protection.	Useful in beverage, emulsion, and lipid delivery systems.	[20,61,66,67]
Chitosan	Polyelectrolyte interaction and coating formation	Supports coatings and may prolong surface activity.	Interesting for preservation films and shelf-life extension studies.	[20,70]
Other proteins (e.g., lysozyme)	Heteroprotein co-aggregation or coacervation	Combines iron sequestering and lytic/foaming functions.	Promising for multifunctional foams and interfacial delivery systems.	[59,63,64,65]

Note: Interaction consequences are expected to depend on pH, ionic strength, ligand competition, and matrix composition. Abbreviations: EGCG, epigallocatechin gallate; Fe^3+^, ferric iron; OVT, ovotransferrin; Zn^2+^, zinc ion.

**Table 5:** Evidence-graded application scenarios for OVT in foods and food-inspired delivery systems.

Scenario	Structured Evidence Tier	Potential Benefit	Key Limitation Before Industrial Application	References
Antimicrobial preservation	Level 2–3	Natural bioactive complementary to coatings or interfacial systems.	Need validation in real foods, shelf-life studies, and sensory assessment.	[57,72,78,79]
Iron-fortified foods	Level 2	Food-compatible iron binding and possible transport-related functionality.	Human food studies, safety, and allergenicity data remain limited.	[8,23,34]
Gut/barrier support formulations	Level 2 with limited in vivo support	Preclinical anti-inflammatory and barrier-protective effects.	Evidence is still largely from cell and animal models.	[18,56,75,82]
Emulsion and oleogel delivery systems	Level 3	Improved interfacial stability and protection of hydrophobic actives.	Scale-up, storage stability, and flavor neutrality must be addressed.	[22,61,64,67]
Functional beverages/liquid foods	Level 3	Fibrillar or complexed OVT can modulate viscosity and improve phytochemical retention.	Matrix compatibility and consumer acceptance need study.	[24,25]
Special population foods	Level 1–2	Conceptually relevant for older adults or compromised gut function.	Clinical efficacy, dose ranges, egg allergen labeling, and risk–benefit justification remain insufficient.	[18,43,81,82]

Note: The evidence levels in this table were assigned according to study type, consistency across independent studies, and the degree of validation in food systems. Level 1 denotes purified protein mechanistic studies; Level 2, simulated digestion, cell, or simplified biological models; Level 3, model food or formulation validation; and Level 4, real food, shelf-life, or in vivo/human evidence directly supporting the claimed use. For food positioning, cell-only or animal-only findings were, therefore, not treated as equivalent to formulation or real food validation. The table is intended as a structured evidence readiness map rather than a quantitative meta-analysis because the underlying studies differ substantially in model system, endpoint definition, and matrix complexity. Abbreviations: OVT, ovotransferrin.

## Data Availability

No new data were created or analyzed in this study. Data sharing is not applicable to this article.
